# CD8^+^ T cells promote tubule-interstitial damage in malaria-induced acute kidney injury

**DOI:** 10.3389/fcimb.2025.1561806

**Published:** 2025-06-30

**Authors:** Douglas Esteves Teixeira, Sarah Aparecida dos Santos Alves, Alessandro Sá Pinheiro, Leandro Souza Silva, Rodrigo Pacheco Silva-Aguiar, Diogo Barros Peruchetti, Tatiana Almeida Pádua, Mariana Conceição Souza, Celso Caruso-Neves, Ana Acacia Sá Pinheiro

**Affiliations:** ^1^ Instituto de Biofísica Carlos Chagas Filho, Universidade Federal do Rio de Janeiro, Rio de Janeiro, Brazil; ^2^ Department of Medicine, Hematology and Oncology, University Hospitals Cleveland Medical Center, Case Western Reserve University, Cleveland, OH, United States; ^3^ Division of Infectious Diseases and Immunology, Department of Medicine, University of Massachusetts Chan Medical School, Worcester, MA, United States; ^4^ Departamento de Fisiologia e Biofísica, Instituto de Ciências Biológicas, Universidade Federal de Minas Gerais, Minas Gerais, Brazil; ^5^ Instituto de Tecnologia em Fármacos, Fundação Oswaldo Cruz, Rio de Janeiro, Brazil; ^6^ Rio de Janeiro Innovation Network in Nanosystems for Health, Rio de Janeiro, Brazil; ^7^ National Institute of Science and Technology for Regenerative Medicine, Rio de Janeiro, Brazil

**Keywords:** malaria, tubule-interstitial injury, acute kidney injury, *Plasmodium*, CD8+ T cells, host defense, host-pathogen interaction

## Abstract

**Introduction:**

Malaria acute kidney injury (MAKI) is associated with severe malaria and correlates with poor prognosis and death of patients infected with Plasmodium falciparum. The pathogenesis of MAKI is not completely understood but some hypotheses are well recognized. Host–parasite interactions lead to mechanical obstruction, disorders in the renal microcirculation, and immune-mediated glomerular injury. We investigated the influence of CD8⁺ T cells in the pathogenesis of malaria-induced renal disease.

**Methods:**

To assess the role of T lymphocytes in MAKI pathogenesis, we used adoptive transfer; antibody-driven CD8^+^ T cells depletion and treatment with FYT720.

**Results:**

The transference of total T cells isolated from malaria-infected donor mice into naive recipient animals reproduced kidney tubule-interstitial damage without affecting glomerular function. It was associated with increased accumulation of CD8^+^ T cells in the kidneys of recipient mice. The selective depletion of CD8^+^ T cells in infected animals resulted in protection from tubular injury, although glomerular alterations still occurred. Finally, we evaluated FTY720, an immunomodulatory drug that sequesters T cells in lymphoid organs and limits their migration, as a potential therapeutic strategy. Treatment with FTY720 prevented the development of proteinuria and the increase in the urine to creatinine ratio. Moreover, FTY720 reduced urinary γ-glutamyl transferase (γ-GT) levels, a marker of tubular injury, but did not alter plasma urea and creatinine, two markers of glomerular function.

**Discussion:**

Our results add new knowledge demonstrating that CD8^+^ T cells have a specific role in tubule-interstitial injury pathology during MAKI.

## 1 Introduction

Malaria is a mosquito-borne infectious disease caused by parasites from the genus *Plasmodium*. In 2022, 249 million people were infected worldwide resulting in 608,000 deaths (World Health Organization, 2023). The clinical presentation of malaria ranges from uncomplicated – characterized by cyclic fever, headache, and fatigue – to severe malaria, commonly caused by *Plasmodium falciparum*. Severe disease is described as a life-threatening disease with the occurrence of different pathologies such as severe anemia, respiratory syndrome, cerebral malaria, and malaria-induced acute kidney injury (MAKI) (White, 2022). MAKI is defined as a rapid and abrupt decline in kidney function and is emerging as one of the most critical complications of severe malaria, frequently associated with poor prognosis and death of patients with *P. falciparum* infection (Conroy et al., 2016; Katsoulis et al., 2021). MAKI can progress as mild proteinuria or as severe azotemia associated with metabolic acidosis, and it may occur as a component of multi-organ dysfunction or as a unique pathology (Mishra and Das, 2008). According to the Kidney Disease: Improving Global Outcomes (KDIGO) definition, the incidence of pediatric MAKI may correspond to 24-59% in children with severe malaria, notably higher than the established by WHO (Batte et al., 2021).

The pathogenesis of MAKI remains to be fully elucidated. Previous reports support the hypothesis that

it results from a combination of parasite and host factors, including the host immune response (Barsoum, 2000; Silva et al., 2018). The contribution of the host immune response has been well addressed in cerebral malaria, where CD8^+^ T cells play a pivotal role. In experimental cerebral malaria (ECM), CD8^+^ T cells are activated in the spleen and subsequently migrate to the brain, where they recognize parasite antigens presented by cross-presenting endothelial cells (Howland et al., 2013; Swanson et al., 2016). A similar mechanism has been observed in malaria-induced lung damage (Claser et al., 2019), but little is known about these processes in the context of MAKI. Kidney damage during malaria infection involves the glomerular deposition of immune complexes associated with mononuclear cell infiltration, promoting glomerular injury (Barsoum, 2000; Nguansangiam et al., 2007). Additionally, interstitial infiltration of immune cells has been shown and correlates with the development of tubule-interstitial injury. Sinniah and Lye reported interstitial inflammatory cell infiltration in tubular segments, including macrophages and T lymphocytes, but not CD4^+^ T cells (Sinniah and Lye, 2000). T cell-mediated kidney damage is well characterized in other conditions, such as tubular nephrite, subclinical acute kidney injury (subAKI), and adriamycin-induced focal segmental glomerulosclerosis (Wang et al., 2001; Waeckerle-Men et al., 2007; Peruchetti et al., 2020). These observations suggest that malaria-activated T cells could contribute to the kidney damage observed in severe malaria.

Here, we used the ECM model to investigate the role of T cells in the development of MAKI. By abrogating the effector function of CD8^+^ T cells, we demonstrated their involvement in inducing tubule-interstitial damage in the kidneys, with no significant impact on glomerular dysfunction. Our results contribute to the knowledge on the role of immune cells in the pathogenesis of MAKI.

## 2 Materials and methods

### 2.1 Ethical statement

All animal handling procedures were approved by the Institutional Ethics Committee of the Federal University of Rio de Janeiro (protocol number A15/22-008-18) and were performed in accordance with the National Institutes of Health (NIH) Guide for the Care and Use of Laboratory Animals.

### 2.2 Mice

Male C57BL/6 mice aged 6–8 weeks were obtained from the Animal Care Facility of the Health Science Center of the Federal University of Rio de Janeiro (UFRJ), Rio de Janeiro, Brazil. Male C57BL/6 mice with GFP controlled by actin promotor (C57BL/6-GFP mice), of the same age, were kindly provided by Dr. Marcelo Bozza, from the Laboratory of Inflammation and Immunology at the Federal University of Rio de Janeiro and were specifically used to analyze the homing of T cells to renal tissue. The animals were maintained in an air-conditioned environment (22°C–24°C) with controlled 12:12 light/dark cycle and free access to standard chow and filtered water. During renal function data collection, the animals were kept in metabolic cages.

### 2.3 Murine malaria model

The GFP-expressing *Plasmodium berghei* ANKA (PbA) parasite used in this study was kindly provided by Dr. Mariana Conceição Souza, Laboratory of Applied Pharmacology, Institute of Drug Technology, Oswaldo Cruz Foundation, Rio de Janeiro, Brazil. The parasite was maintained and propagated as per standard procedures in our laboratory, with periodic passages through C57BL/6 mice to ensure its viability and infectivity. The specific passage cycle and maintenance conditions were consistent with previously established protocols in our research group (Silva-Filho et al., 2013). ECM was established as previously described (Pontes et al., 2021). Briefly, C57BL/6J mice received intraperitoneal (IP) injection of 1 × 10^6^ PbA-infected red blood cells (iRBCs) obtained from mice of the same background. Parasitemia in peripheral blood samples was monitored on days 3, 4, and 5 post infection by flow cytometry and was validated by optical microscopy (data not shown) using hematologic stained thin blood smears obtained from the tail of each animal. Flow cytometry was used as the primary method to assess parasitemia in most experiments. Optical microscopy was specifically used to quantify parasitemia in C57BL/6-GFP donor animals for experiments analyzing T cell migration to the kidneys. The results were expressed as the percentage of infected cells. The percentage parasitemia corresponded to the number of iRBCs divided by the total number of RBCs in all blinded-counted fields, multiplied by 100.

### 2.4 FTY720 treatment

Mice were randomly divided into 4 groups: (1) non-infected mice (naive), (2) non-infected mice treated with FTY720 (FTY720), (3) mice infected with PbA (PbA-infected), and (4) mice infected with PbA that received treatment with FTY720 (PbA-infected + FTY720). Treatment with 0.3 mg/kg FTY720 (SML0700, Sigma-Aldrich, St Louis, MO) started 1 day after infection with PbA. FTY720 was administered daily (IP) until day 4 post infection. Non-treated groups received the saline vehicle as controls. On day 5, mice were euthanized to collect blood and kidney samples for analysis.

### 2.5 Adoptive transfer of splenic T cells

Splenocytes were isolated from naive or infected mice (donor mice) on day 5 after infection and treated with an ACK solution (155 mM NH₄Cl, 12 mM NaHCO_4_, and 0.1 mM EDTA) for 1 minute to induce red blood cell lysis. The cells were then washed with PBS supplemented with 10% FBS. An aliquot of the solution was analyzed by flow cytometry to verify the efficiency of erythrocyte lysis, confirming the absence of GFP-positive signals from the parasite. Cell suspensions were passed through sterile nylon wool columns and maintained for 45 minutes in a humidified atmosphere with 5% CO_2_ at 37°C to collect the effluent population of enriched T cells, achieving a purity of 96% CD3^+^ gated cells (data not shown). Cell viability was assessed using 0.25% trypan blue dye exclusion and was greater than 92%. In some groups of animals, the T cells were then labeled with a 1 µM solution of CFSE (C34554, Invitrogen, Waltham, MA) following the manufacturer’s instructions, and 1 × 10_6_ cells were injected intravenously into naive acceptor mice (Silva-Filho et al., 2013), to analyze the migration of cells to malaria target organs. Three days after adoptive transfer, the spleen, lungs, kidneys, and brain were collected from acceptor mice for analysis of CFSE-labeled cells by flow cytometry (FACScalibur, BD Biosciences, San Jose, CA). In addition, T cell migration to the kidney was confirmed using C57BL/6 mice expressing GFP infected with PbA, as donors. T cell migration was quantified as a percentage of CD3^+^/CFSE^+^ or CD3^+^/GFP^+^ cells in relation to the total CD3+ cells contained in each organ analyzed. The groups that received unlabeled T cells were kept in metabolic cages to determine renal functional parameters, structural analysis, and quantification of cytokines and renal T cells.

### 2.6 Antibody-driven CD8^+^ T cell depletion

Mice infected with PbA received 25 mg/kg of anti-CD8 (catalog no. 553026; BD Pharmingen, San Diego, CA) or control isotype rat IgG on days 2 and 3 after infection. Depletion in the spleen and kidney was assessed by flow cytometry on day 5 post infection.

### 2.7 Kidney injury RNA seq dataset analysis

Gene expression analysis was performed using the RNA sequencing profile from the Gene Expression Omnibus (GEO) database. The databases investigated were: GSE253390 (Ramos et al., 2024), GSE44925 (Fähling et al., 2013), GSE247727 (Heruye et al., 2024), GSE183455. The expression of the genes of interest were normalized using the R statistical environment, version 4.0.1. The correlation graphs were generated based on the differences in the mean values of the kidney injury group compared to those of their respective controls.

### 2.8 Renal function

Renal function was accessed according to previously published papers (Peruchetti et al., 2020; Teixeira et al., 2020). 24 h-urine samples were obtained in metabolic cages to determine urinary protein excretion (catalog no. 36), γ-GT activity (catalog no. 105–2/50), urinary creatinine (catalog no. 35-100), using commercial kits, according to the manufacturer’s instructions (Labtest Diagnóstica SA, Lagoa Santa, Brazil). Plasma samples were used to determine the concentration of creatinine (catalog no. 35-100) and blood urea nitrogen (catalog no. 27-500) (Labtest Diagnóstica). The mass of protein excreted in 24 h, creatinine clearance (CCr), and the ratio of urinary protein to urinary creatinine (UPCr) were calculated.

### 2.9 Histological analysis

The histology studies were performed as described previously (Portella et al., 2013; Peruchetti et al., 2020; Teixeira et al., 2020). Briefly, the mice were perfused with saline and 4% paraformaldehyde using a peristaltic pump at a flow rate of 10 mL/min. The kidneys were removed, fixed in Gendre’s solution (80% saturated picric acid in ethanol, 15% formalin, 5% glacial acetic acid) for 24 hours, then in 10% buffered formalin for 48 hours, and subsequently embedded in paraffin. 5-μm-thick kidney sections were stained with periodic acid-Schiff for histomorphometry analysis and 8-um-thick sections were stained with Picrosirus red to evaluate collagen deposition. Fifteen images were randomly acquired using a Nikon 80i eclipse microscope (Nikon, Japan). Tubular alterations were assessed by quantifying the interstitial space and interstitial cellularity. For the glomerulus, glomerular cellularity and Bowman’s capsule space were analyzed. The interstitial space was calculated as the ratio between the interstitial area per field and the total area of the field. Interstitial cellularity was determined by quantifying the number of cells within the interstitial space per field. The Bowman’s capsule space was calculated as the ratio of the Bowman’s space area to the total glomerular area. All parameters were analyzed with Image-Pro Plus software (Media Cybernetics, Rockville, MD). The analysis was performed in blind fashion.

### 2.10 Immunoblotting analysis

Immunoblotting was performed as published previously (Caruso-Neves et al., 2005; Peruchetti et al., 2020; Teixeira et al., 2020). Renal cortex samples were homogenized in a solution containing 10 mM HEPES-Tris (pH 7.6), 250 mM sucrose, 2 mM EDTA, 1 mM PMSF, and 1× protease inhibitor cocktail (catalog no. I3786, Sigma-Aldrich). For protein analysis, total proteins were measured in renal cortex samples and creatinine in urine samples. The samples were diluted in 250 mM sucrose and mixed with sample buffer (125 mM Tris-HCl pH 6.8, 4% SDS, 20% glycerol, 10% β-mercaptoethanol, and 0.01% bromophenol blue) to achieve a final concentration of 2 mg/mL for renal cortex proteins and 1 mg/mL for creatinine in urine samples. 40 µg of cortex homogenate proteins and urine samples normalized according to the urinary creatinine levels were resolved on SDS-PAGE and transferred to PVDF membranes. Membranes were incubated with goat anti-mouse-perforin-1 primary antibody (sc-7417; Santa Cruz Biotechnology, Dallas, TX), rabbit anti-albumin (ab207327, Abcam, Cambridge, MA, USA), mouse anti-β-2-microglobumin (SAB4700014, Sigma-Aldrich, St. Louis, MO, USA) or mouse anti-KIM-1 (ab233720, Abcam, Cambridge, MA, USA) primary antibodies followed by incubation with their respective HRP-conjugated IgG secondary antibodies, according to the manufacturer’s instructions. ECL Prime (GERPN2236, Cytiva, Marlborough, MA) was used to detect proteins of interest. Images were obtained using the Image Quant LAS 4000 Image processing system (GE Healthcare Life Sciences, Pittsburgh, PA). The densitometry values of the proteins of interest were corrected by the densitometry of the β-actin (catalog no. 4967; Cell Signaling Technology, Danvers, MA) or GPDH (catalog no. 2118L; Cell Signaling Technology, Danvers, MA) used as loading control.

### 2.11 Renal cytokines

The level of renal cortical cytokines was determined as described previously (Landgraf et al., 2014; Peruchetti et al., 2020; Teixeira et al., 2020). Briefly, the concentrations of IL-17, IL-6, IFN-γ, TNF, and IL-4 in the homogenate samples were quantified by ELISA (BD Biosciences) and the values were normalized to total protein.

### 2.12 Flow cytometry

T cells in the kidney and spleen were assessed as described previously (Peruchetti et al., 2020; Teixeira et al., 2020; Pontes et al., 2021). Briefly, thick sections of kidney were cut in RPMI Media 1640 supplemented with 0.05 mM 2-mercaptoethanol, 2 mM L-glutamine, 1 mM sodium pyruvate, and 10 mM HEPES. The sections were added to a solution containing 0.1% collagenase type IV (C4-22, Sigma-Aldrich, St Louis, MO) for 60 min at 37°C, and samples were then filtered through cell strainers. In the next step, the cells were treated with ACK solution to lyse red blood cells and washed with PBS supplemented with 10% serum.

The cells were incubated with the following antibodies for surface markers: PeCy5.5-conjugated rat IgG2a anti-murine CD8 (45-0081-80, Thermo Fisher Scientific, Waltham, MA), PE-conjugated rat IgG2b anti-mouse CD4 (clone GK1.5; 11-0041-82, Thermo Fisher Scientific), and FITC-conjugated rat IgG1 anti-mouse CD45 (11.0452-82, Thermo Fisher Scientific). For T cell migration experiments, the cell suspension was incubated with a PeCy5.5-conjugated hamster IgG1 anti-murine CD3 antibody (145-2C11, eBioscience, San Diego, CA, USA). Migration was quantified by determining the percentage of CD3+/CFSE+ cells relative to the total number of CD3+ cells in the organ of interest. The cells were then washed twice and analyzed in a BD FACSCalibur cytofluorometer (BD Biosciences) using CellQuest software.

### 2.13 Statistical analysis

All box plot graphs are presented as median (interquartile range), line graphs are presented with mean and standard error. GraphPad Prism 7 (version 8, GraphPad Software, San Diego, CA, USA; www.graphpad.com) was used for the statistical analysis. The normality distribution was assessed by the Shapiro-Wilk test. Unpaired Student’s t-test was used for comparison between two groups and one-way analysis of variance (ANOVA) followed by Tukey’s *post hoc* test was used to compare four groups. P < 0.05 was used to determine statistical significance. N values reflect a compilation of data from multiple experiments. When appropriate, correlation was assessed with the Spearman r coefficient.

## 3 Results

### 3.1 T cells activated during malaria infection have a role in the genesis of renal tubular injury

**Figure 1:**
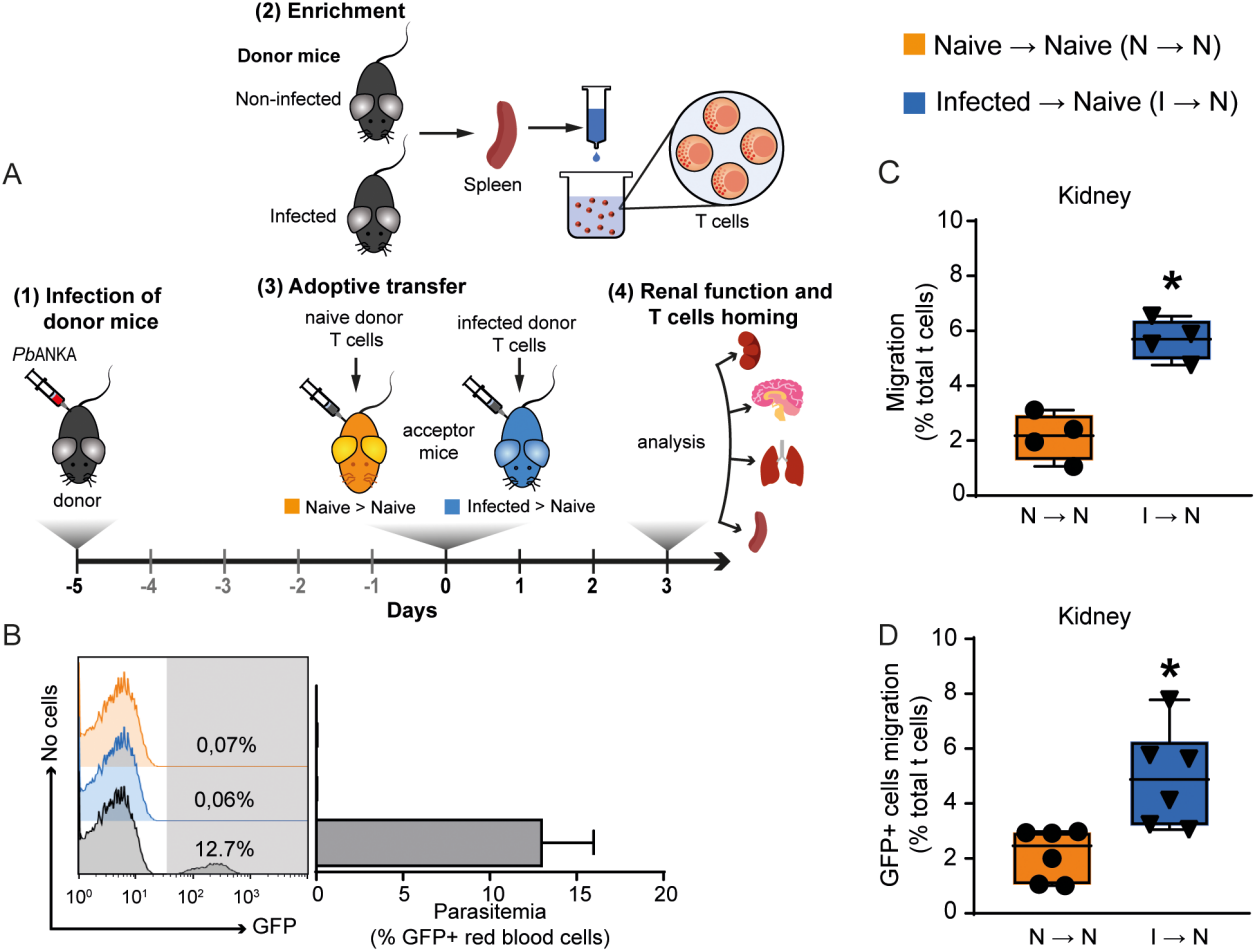
Malaria-responsive T cells migrate to target organs. **(A)** Schematic representation of experimental design. Donor animals were infected 5 days before adoptive transfer. T cells from the donors’ spleens were enriched in wool columns and transferred to naive acceptor animals, generating two groups: acceptors that received T cells from uninfected donors (Naive → Naive); acceptors that received T cells from infected donors (Infected → Naive). On the third day after adoptive transfer, T cell migration was analyzed, and renal function was assessed. **(B)** Parasitemia was observed only in donor infected mice (gray bar) (n = 6–8). **(C)** Migration of malaria-responsive T cells to the kidney by CD3^+^/CFSE ^+^ cells (n = 4). **(D)** To confirm migration to the kidney, C57BL/6-GFP animals, infected or not, were used as donors to perform adoptive transfer (n = 6). Migration is expressed as the percentage of CD3^+^/CFSE^+^ or CD3^+^/GFP^+^ cells relative to the total CD3^+^ cells in the organ of interest. *versus naive → naive group, P < 0.05.

The murine model of severe malaria, induced by infecting C57BL/6 mice with PbA, mimics several aspects of the renal injury seen in humans infected with *Plasmodium falciparum* (Katsoulis et al., 2021). While our group and others have demonstrated glomerular and tubular damage in this model, the role of T cells in this process remains unclear (Abreu et al., 2014; Souza et al., 2015; Silva et al., 2018; Ramos et al., 2019). In the first experimental group, we used adoptive transfer experiments to better investigate the role of T cells in the genesis of renal injury induced by malaria. For this, spleen-derived T cells (1 × 10^6^ cells) from naive or infected mice were harvested on day 5 post-infection (p.i.), labeled with CFSE and transferred to acceptor naive mice. Renal function was evaluated on day 3 post adoptive transfer (**Figure 1A**). Using fluorescence-activated cell sorting (FACS) analysis, we certified that acceptor mice did not develop parasitemia after adoptive transfer, although 12.7% parasitemia occurred in donor infected mice (**Figure 1B**). These results confirmed that there was no contamination with infected erythrocytes in our preparation. The presence of CFSE-labeled T cells in different malaria target organs was evaluated in acceptor mice to determine the percentage of total T cells. T cells obtained from PbA-infected mice were able to traffic to the lung, kidney, spleen, and brain more avidly than those from naive donor mice (**Figure 1C**; **Supplementary Figure 1**). Homing of T cells to the kidney without re-stimuli was confirmed using C57BL/6-GFP mice, infected or not, as donors to perform adoptive transfer (**Figure 1D**).

**Figure 2:**
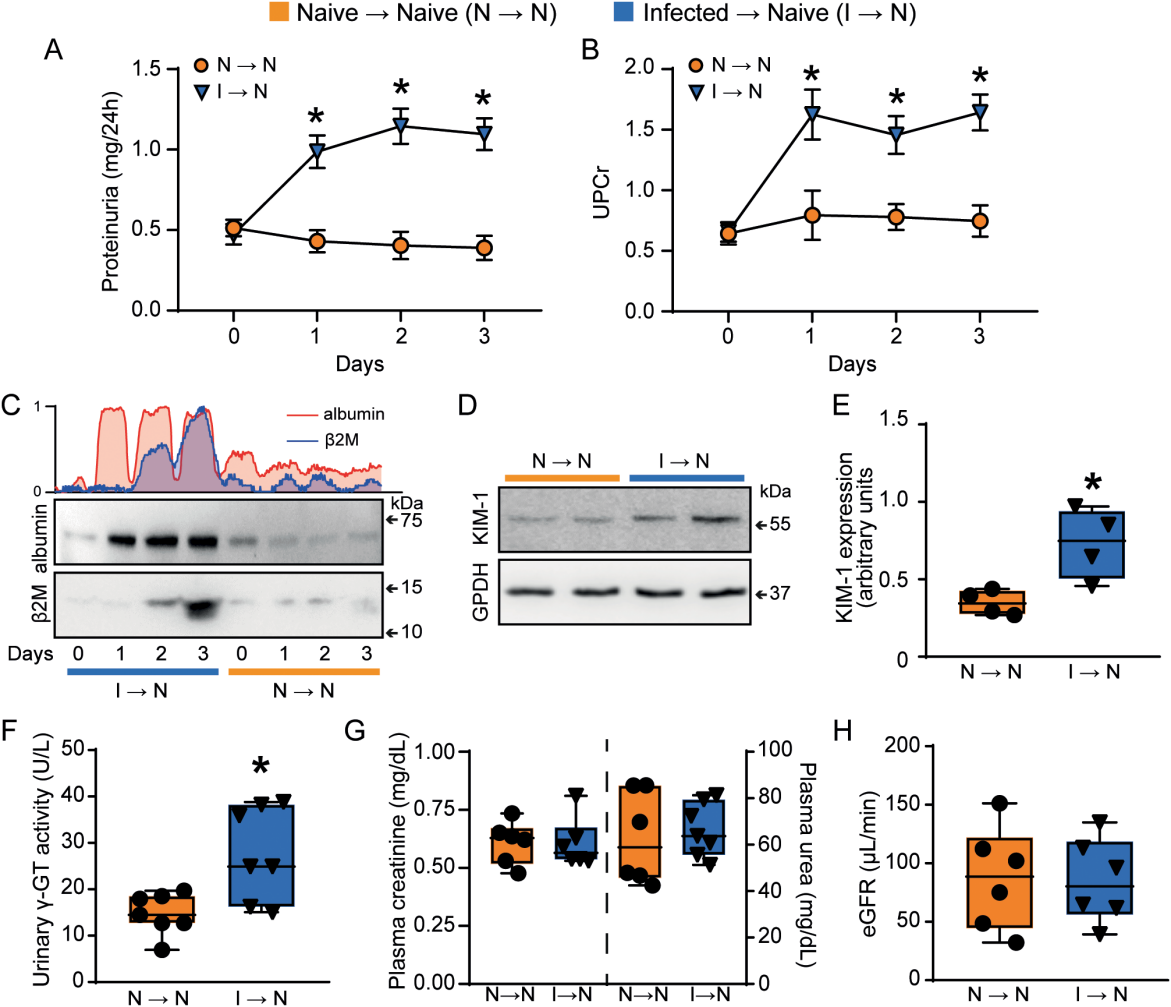
Malaria-responsive T cells induce tubule-interstitial injury. Quantification of urine protein excretion by **(A)** total proteinuria measured in 24h (n = 7) and **(B)** UPCr (n = 7). **(C)** Representative immunoblotting of urinary albumin and β2M (n = 3). **(D, E)** Representative immunoblotting of cortical KIM-1 and quantification of KIM-1 expression. GPDH was used as load control (n=4). **(F)** urinary γ-GT activity (n = 7). Urinary β2M, urinary γ-GT activity and cortical KIM-1 expression were used as tubular injury markers. The glomerular function was assessed using **(G)** plasma creatinine (n = 6), plasma urea (n = 6–7), and **(H)** creatinine clearance (n = 6). *versus naive → naive group, P < 0.05.

**Figure 3:**
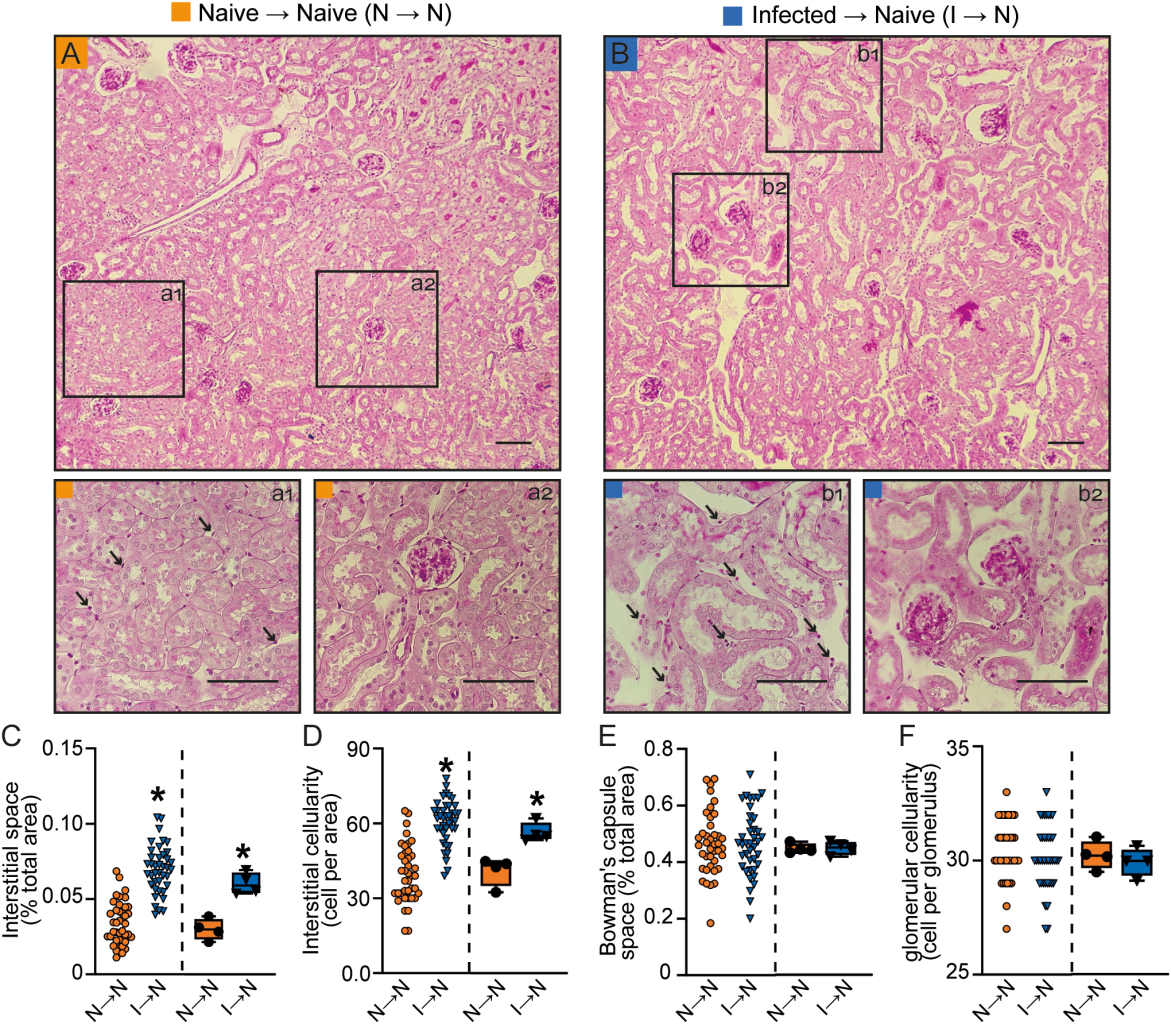
Malaria-responsive T cells induce tubular morphofunctional changes without significant glomerular effects. **(A, B)** Representative images of periodic acid-Schiff (PAS)-stained cortical slices showing the tubular and glomerular regions. Panels a1 and b1 display magnified views of the interstitial region, while a2 and b2 show magnified views of the glomeruli. (scale bar, 150 μm, n = 5). **(C)** Interstitial space and **(D)** interstitial cellularity. **(E)** Bowman’s capsule space and **(F)** glomerular cellularity. The dispersion plots in graphs **(C-F)** (on the left) represent the number of fields analyzed in the tubular and glomerular regions, while the box plots (on the right) show the mean values derived from the different fields for each animal (n = 4). Statistics were performed based on the average value of each animal analyzed. *versus naive → naive group, P < 0.05.

Mice adoptively transferred with T cells from PbA-infected donors (infected → naive) exhibited increased 24-h proteinuria and UPCr, which correlated with elevated urinary albumin levels compared to acceptor mice that received naïve T cells (naive T cells → naive mice) (**Figures 2A–C**). Moreover, T cells from PbA-infected donors promoted an increase in three different tubular injury markers: urinary β2-microglobulin (β2M), cortical KIM-1 and urinary γ-GT activity (**Figures 2C–F**). In contrast, the glomerular function, assessed by plasma urea and creatinine levels, and estimated glomerular filtration rate (eGFR) measured by creatinine clearance, remained unchanged (**Figures 2G, H**). Accordingly, morphofunctional analysis revealed that adoptive transfer of PbA-activated T cells induced an increase in tubule-interstitial space and tubule-interstitial cellularity (infected → naive) compared to mice receiving naive T cells (naive → naive) (**Figures 3A–D**). No significant changes were observed in Bowman’s capsule space or glomerular cellularity when both groups were compared (**Figures 3A, B, E, F**).

Consistently, a proinflammatory profile was observed in the kidney of naive acceptor mice that received T cells from PbA-infected donors (infected → naive) when compared to those adoptively transferred with naive T cells (naive → naive mice). We observed an increase in interleukin (IL)-17, IL-6, and interferon-γ (IFN-γ), with a more prominent increase for IFN-γ and IL-6. No difference in the levels of tumor necrosis factor (TNF) or IL-4 were detected (**Supplementary Figures 2A–F**). These data support the notion that T cells activated during PbA infection have a specific role in the tubular injury observed in MAKI without affecting the glomerular component.

**Figure 4:**
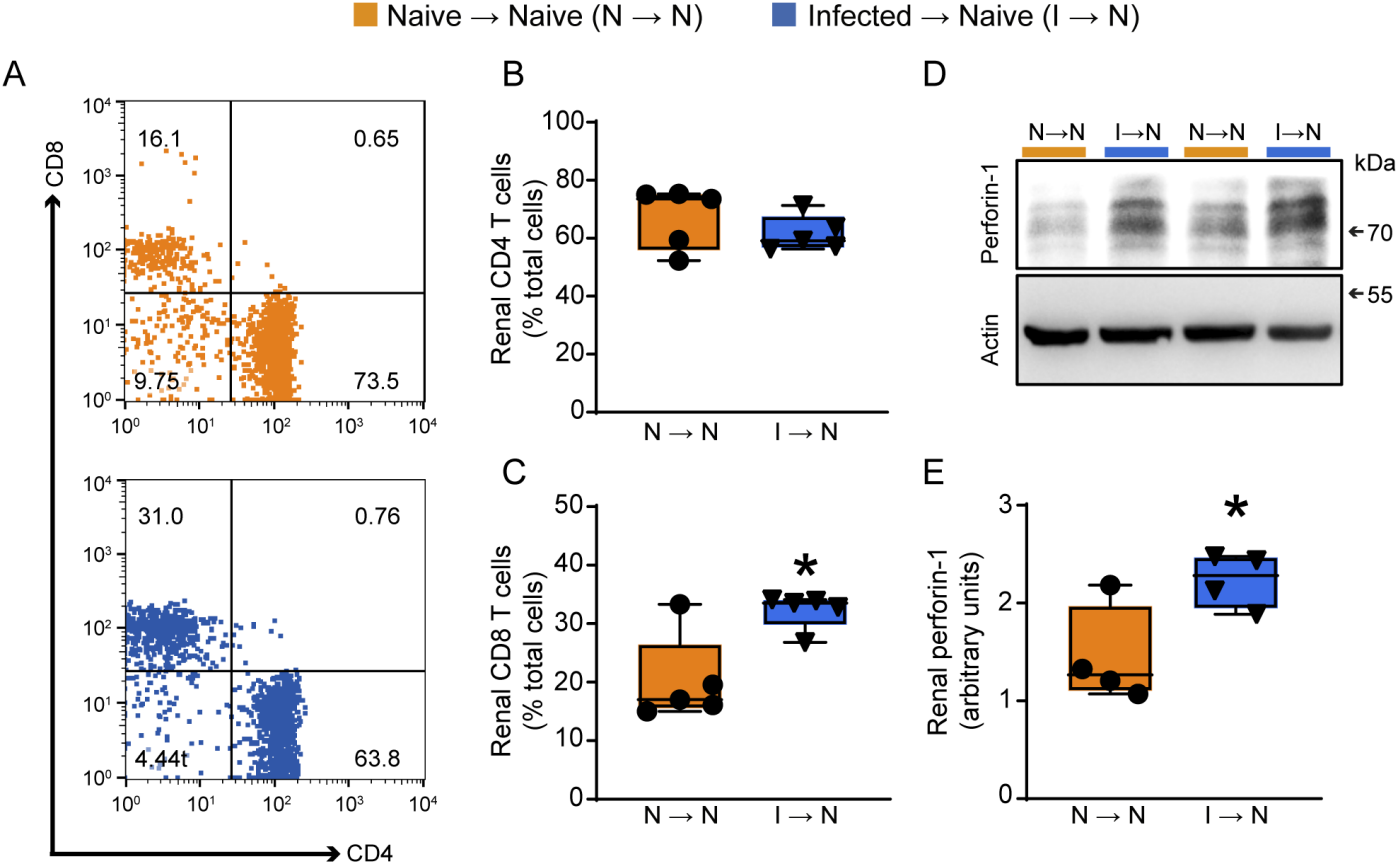
Adoptive transfer of malaria-responsive T cells induces an increase in CD8^+^ T cells population in renal tissue. **(A)** Dot-plot representing the percentage of CD4 and CD8^+^ T cells in total CD45^+^ renal cells of the naive → naive (orange) and infected → naive (blue) groups. **(B)** Quantification of the percentage of CD4^+^ (n = 5) and **(C)** CD8^+^ T cells (n = 5) in the kidney. **(D)** Representative immunoblotting of cortical perforin-1 expression in renal tissue. **(E)** Quantification of perforin-1 expression in renal tissue (n = 4). *versus naive → naive group, P < 0.05.

In the next step, we sought to determine the subset of T lymphocytes involved in the establishment of tubular dysfunction. For this, 3 days after adoptive transfer, mice were euthanized, and the total T cells were harvested from the kidney. Cells were gated to CD3 to determine the percentage of CD4^+^ or CD8^+^ T cells. We found no difference in the frequency of CD4^+^ T cells between groups (naive → naive or infected → naive) (**Figures 4A, B**). However, the frequency of CD8^+^ T cells was increased in naive mice that received T cells from PbA-infected donors (infected → naive, **Figures 4A, C**) compared to controls (naive → naive). Also, we observed increased renal perforin-1 expression compared to the control group that received naïve cells, as revealed by western blotting analysis (**Figures 4D, E**).

To confirm that the increase in CD8^+^ T cells in the kidney was due to enhanced migration rather than a higher frequency of this cell type in the transferred population, we analyzed CD4^+^ and CD8^+^ T cells in the peripheral blood of recipient mice (**Supplementary Figures 3A–C**). No significant changes were observed in the peripheral frequencies of either CD4^+^ or CD8^+^ T cells (**Supplementary Figures 3B, C**). Furthermore, we calculated the kidney-to-blood ratio for both CD4^+^ and CD8^+^ T cells. While no changes were observed in the CD4^+^ T cell ratio, we found an increased kidney-to-blood ratio for CD8^+^ T cells (**Supplementary Figures 3D, E**). These results suggest the specific involvement of the CD8+ T cells subset in the tubule-interstitial injury observed.

### 3.2 CD8^+^ T cells play a role in malaria-induced tubule-interstitial injury

**Figure 5:**
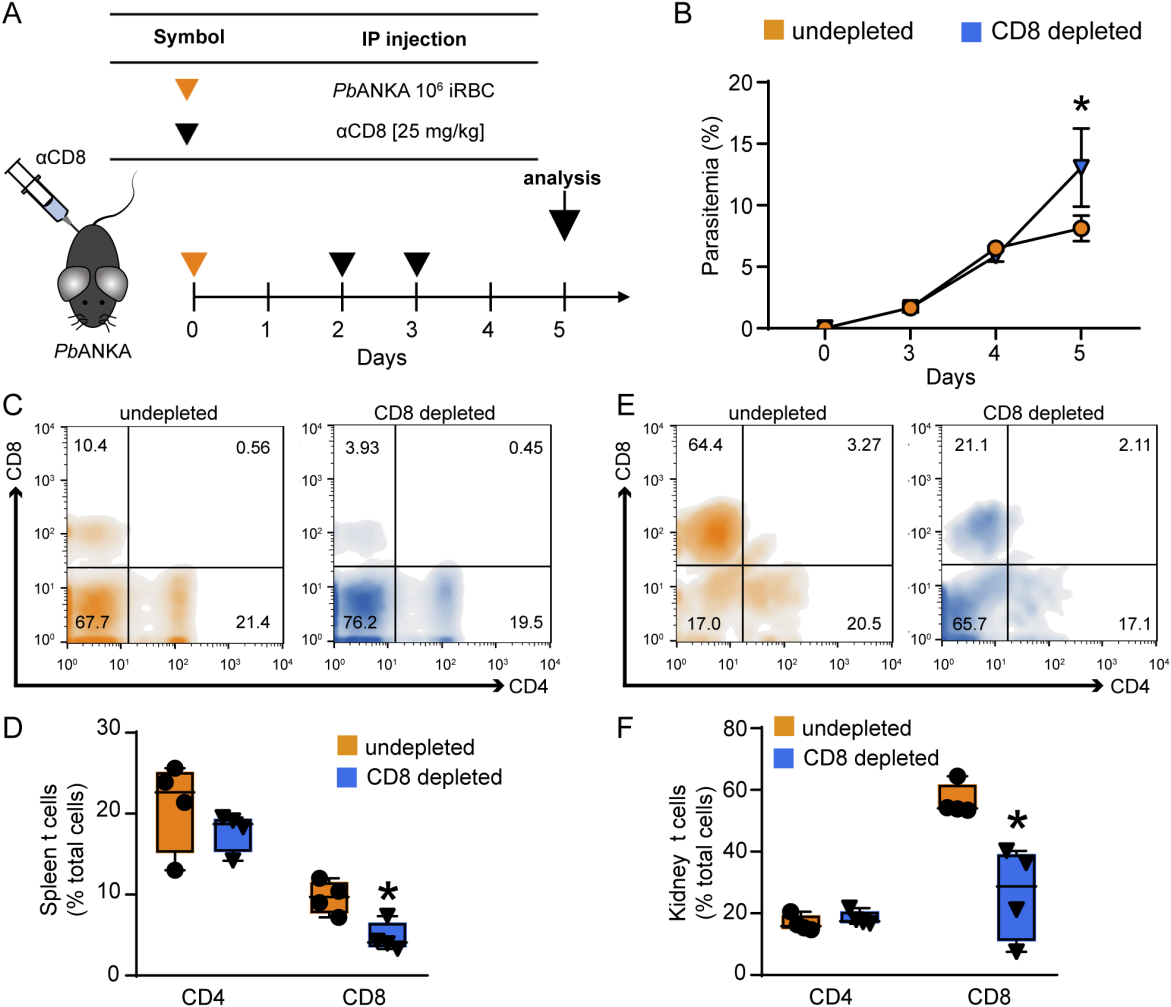
Depletion of CD8^+^ T cells induces an increase in parasitemia but does not change the percentage of CD4 T cells. **(A)** Representative scheme of CD8^+^ T cell depletion. Animals were infected with 10^6^ iRBCs and treated with anti-mouse CD8 antibody on the day 2 and 3 post infection. The analyses were performed on day 5. **(B)** Peripheral parasitemia was assessed daily (n = 4). **(C)** Histogram representing the percentage of CD4^+^ and CD8^+^ T cells in CD45^+^ cells of the spleen. **(D)** Quantification of the percentage of CD4^+^ and CD8^+^ T cells in spleen (n = 4). **(E)** Histogram representing the percentage of CD4^+^ and CD8^+^ T cells in CD45^+^ cells of the kidney. **(F)** Quantification of the percentage of CD4^+^ and CD8^+^ T cells in spleen (n = 4). *versus undepleted group, P < 0.05.

To target the specific pathogenic role of CD8^+^ T cells in MAKI, mice were treated with anti-CD8 antibody (i.p.) on days 2 and 3 post infection to deplete CD8^+^ T cells (**Figure 5A**). Anti-heavy chain IgG was used as control. Depletion of CD8^+^ T cells did not change the percentage of CD4^+^ T cells, but it significantly reduced the CD8^+^ T cell population in the kidney and spleen of infected mice, as assessed on day 5 p.i. (**Figures 5C–F**). Interestingly, this treatment resulted in increased parasitemia on the day 5 p.i., highlighting the role of CD8^+^ T cells in controlling the parasite load (Imai et al., 2015; Safeukui et al., 2015) (**Figure 5B**).

**Figure 6:**
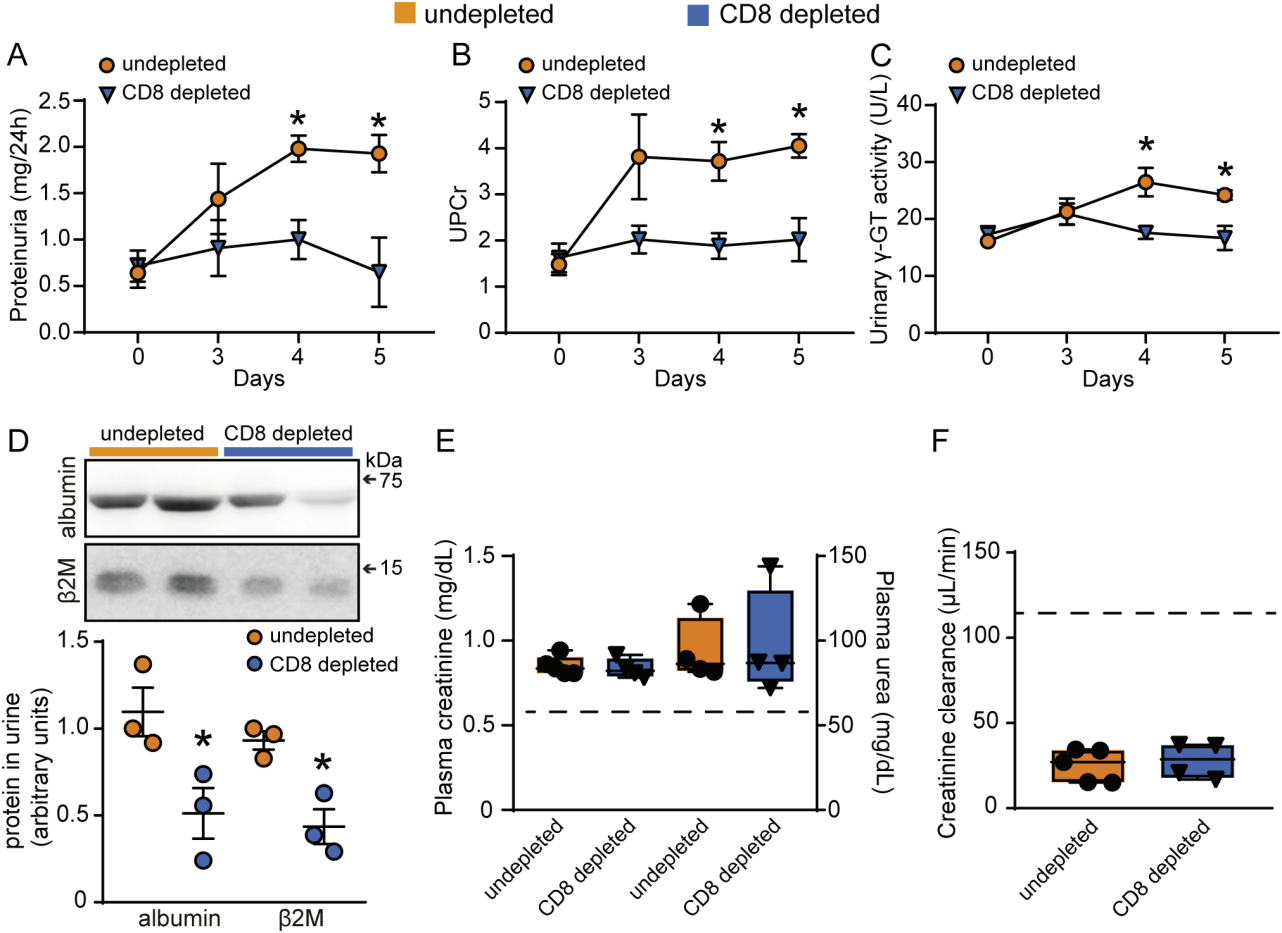
Depletion of CD8^+^ T cells protects against tubular injury induced by PbA infection. Quantification of urine protein excretion by **(A)** 24-h proteinuria and **(B)** UPCr in the undepleted and CD8 depleted groups (n = 4). **(C)** Analysis of γ-GT enzyme activity in urine samples (n = 4). **(D)** Representative image of the urinary excretion of albumin and β2M and quantification graph (n=3). **(E)** Quantification of plasma levels of creatinine and urea (n = 4). **(F)** Estimated glomerular filtration rate assessed by creatinine clearance (n = 4). Dashed line indicates the levels of the untreated naive group. *versus undepleted group, P < 0.05.

Regarding renal function, depletion of CD8^+^ T cells prevented the increase in 24-h proteinuria, UPCr as well as albuminuria, measured by western blotting (**Figures 6A, B, D**). Moreover, urinary γ-GT activity and β2M, both increased during infection, were remarkably reduced following CD8^+^ T cell depletion (**Figures 6C, D**). On the other hand, malaria-induced glomerular damage, assessed by plasma creatinine, plasma urea, and eGFR, was not changed (**Figures 6E, F**). These results reinforce the notion that CD8^+^ T cells, activated during malaria, migrate to the kidneys and specifically induce tubule-interstitial injury, a key component in the development of MAKI.

### 3.3 The gene expression profile during MAKI has similarities to other models of kidney injury

**Figure 7:**
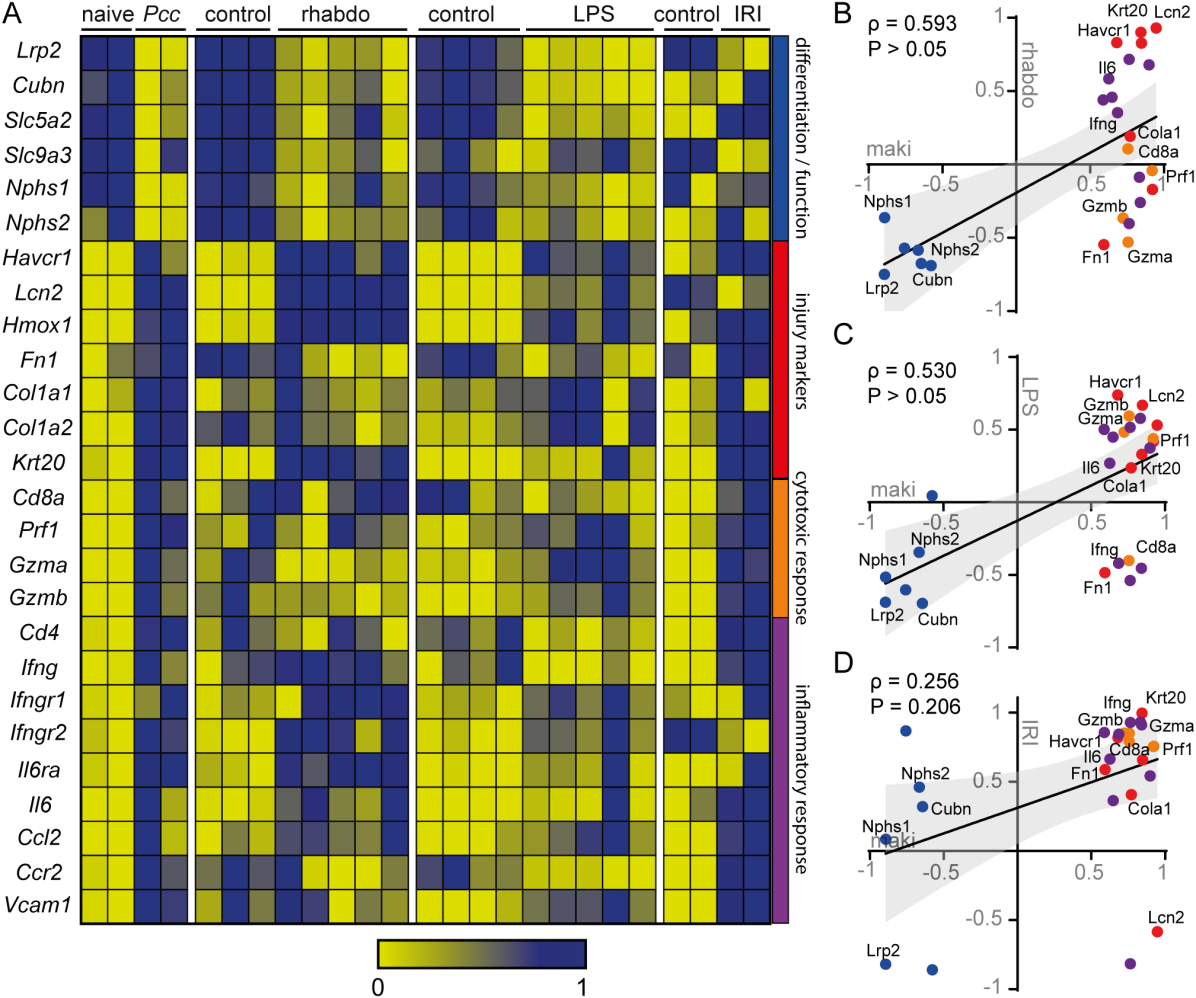
The gene expression profile of the MAKI model correlates with other models of kidney injury **(A)** Heatmap graph of RNAseq database of murine kidney injury models of: MAKI, rhabdomyolysis, endotoxemia and ischemia and reperfusion. The gene profile was selected based on genes involved in the function and differentiation of tubules and glomerulus, injury markers, cytotoxic response and inflammatory process. Correlation graph between MAKI and rhabdomyolysis **(B)**, endotoxemia **(C)** and ischemia and reperfusion **(D)** model, based on data extracted from the RNAseq database used in **(A)** In all representations, genes were normalized between 0 and 1. For graphs B, C and D, the mean of the normalized values ​​of the control was subtracted by the test condition (Naive – Pcc, Control – Rhabdomyolysis, Control – LPS and Control – IRI). The Spearman r correlation coefficient and P value were calculated. P < 0.05 were considered significant.

With the aim of comparing our findings in PbA-induced MAKI with the literature, we analyzed gene expression using an RNA sequencing (RNAseq) database from animals with MAKI induced by *Plasmodium chabaudi* (Pcc) infection (**Figure 7A**). The database revealed that infection with Pcc reduced the expression of genes involved with differentiation and function of glomerular and tubular cells. More specifically, there was a decrease in *Nphs1* and *Nphs2*, which encode nephrin and podocin, key proteins of the glomerular filtration barrier, as well as in *Lrp2* and *Cubn*, which encode megalin and cubilin, respectively, receptors involved in protein reabsorption in the proximal tubule. This was accompanied by an increase in tubular injury markers, such as *Havcr1*, *Col1a1* and *Col1a2* which encode KIM-1 and type 1 and 2 collagen, respectively; markers that were also identified in our study with PbA infection. Moreover, we observed an increase in genes associated with cytotoxic and pro-inflammatory responses, including those encoding CD8 (*CD8a*), perforin-1 (*Prf1*), IFN-γ (*Ifng*, *Ifngr1*, *Ifngr2*) and IL-6 (*Il6*, *Il6r*), all of which were similarly increased in the kidneys of PbA-infected mice.

In addition, we compared the gene expression profiles of MAKI with those of other kidney injury models (**Figure 7A**). Similar to MAKI, kidney injury induced by rhabdomyolysis and endotoxemia also exhibit a decrease in genes involved in the differentiation and function of the glomeruli and tubular segments. Similar results were observed for proximal tubule injury markers *Havcr1* and *Lcn2*, as well as the distal tubule injury marker *Lcn2* and the pro-inflammatory cytokines *Ifng* and *Il6*. In contrast, the ischemia-reperfusion kidney injury model (IRI) did not recapitulate changes in the genes associated with the differentiation and function of tubules and glomeruli, and most kidney injury markers were unaffected. Instead, this model predominantly showed an increase in markers related to extracellular matrix deposition, such as *Fn1*, *Col1a1*, and *Col1a2*, which encode fibronectin and collagen types 1 and 2, respectively. Furthermore, genes involved in cytotoxic and inflammatory responses were significantly elevated in the IRI model, similar to what was observed in the MAKI experimental model.

Recapitulating the similarities and differences in the gene responses across the kidney injury models, we identified a positive correlation between MAKI and the kidney injury models induced by rhabdomyolysis and endotoxemia (**Figures 7B, C**). In contrast, no correlation was found between MAKI and the IRI model (**Figure 7D**). These findings suggest that renal injury induced by rhabdomyolysis and endotoxemia share significant similarities regarding the responses of the genes of interest.

### 3.4 FTY720 ameliorated renal tubular dysfunction during infection with *P. berghei* ANKA

**Figure 8:**
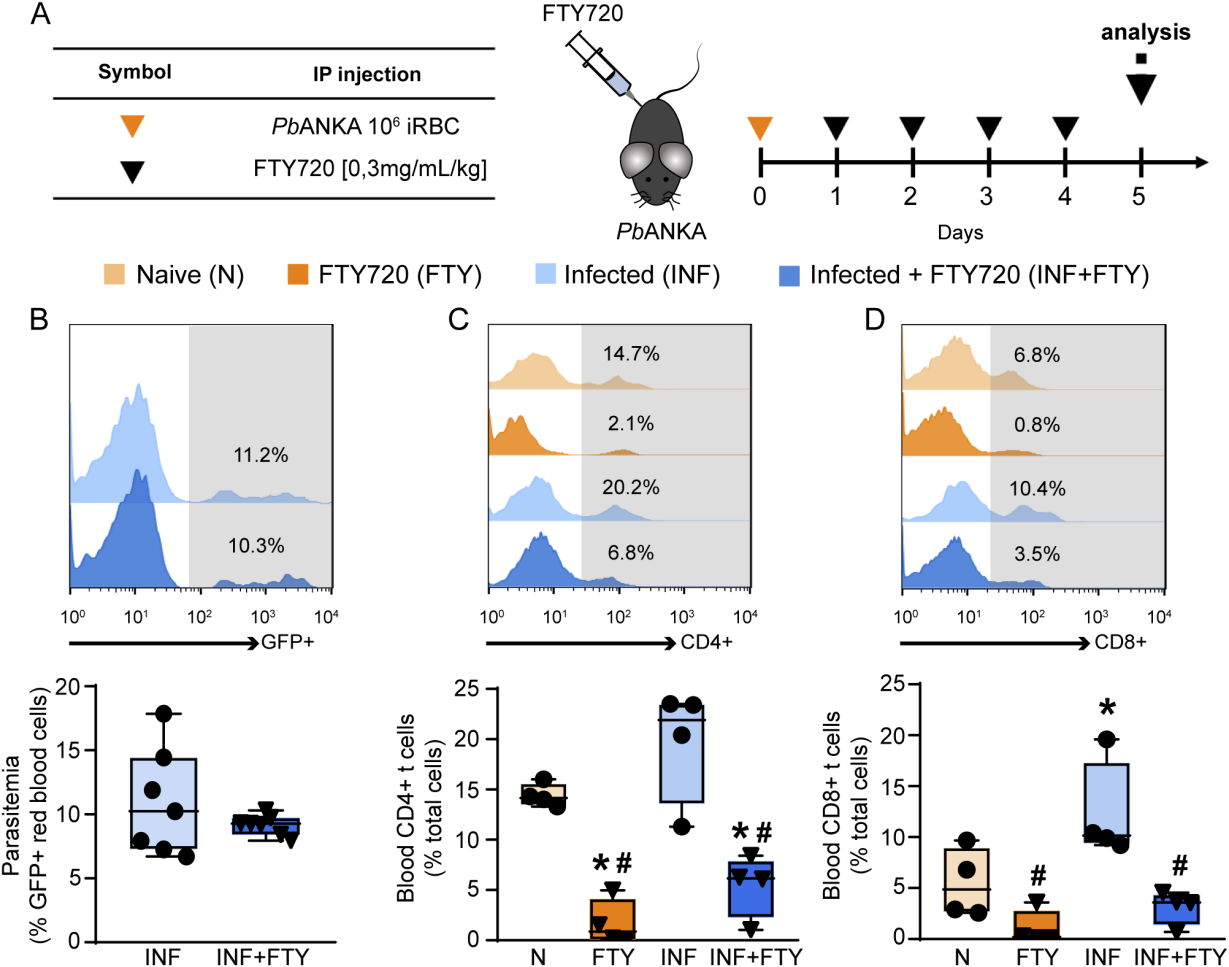
FTY720 induces immunosuppression without affecting parasitemia. **(A)** Scheme for infection with *Pb*A and treatment with FTY720. The animals were infected with 10^6^ infected RBCs (iRBCs) on day 0 and treated from the day 1 to day 4 post infection with FTY720 at a concentration of 0.3 mg/mL/kg. **(B)** Peripheral parasitemia level on day 5 post infection (n = 7). **(C)** FACS analysis of blood CD4^+^ T cells (n = 4) and **(D)** blood CD8^+^ T cells (n = 4). *versus naive group, P < 0.05; versus infected group, P < 0.05.

Given the deleterious role of CD8+ T cells in malaria-induced tubular injury, we aimed to evaluate the effects of FTY720 (fingolimod), an immunomodulatory drug that inhibits the egress of lymphocytes from lymphαoid tissues αnd their activation (Baer et al., 2018), as a potential treatment for MAKI. For this, the animals were infected and treated for 4 consecutive days with 0.3 mg/kg FTY720. Four groups were established (**Figure 8A**): (1) non-infected control mice (naive); (2) non-infected mice treated with FTY720 (FTY720); (3) mice infected with *Plasmodium berghei* ANKA (infected); and (4) mice infected with PbA treated with FTY720 (infected + FTY720).

**Figure 9:**
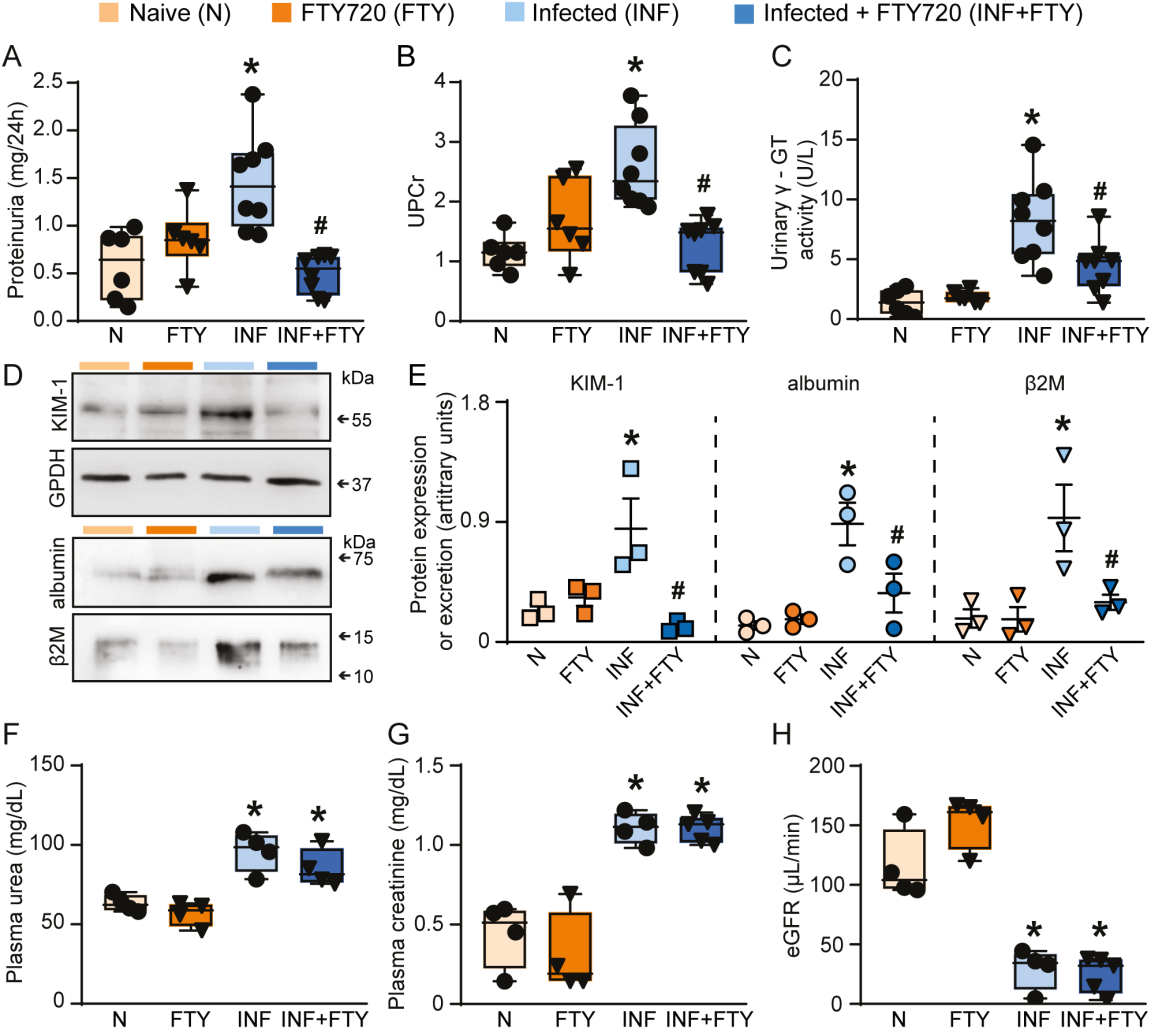
FTY720 treatment ameliorates renal tubular injury but does not affect glomerular damage during PbA infection. Quantification of urine protein excretion by **(A)** 24h proteinuria (n = 6-8) and **(B)** UPCr (n = 6-8). Tubular injury was assessed by **(C)** γ-GT urinary activity (n = 6-8). **(D)** Representative immunoblotting of cortical KIM-1. GPDH was used as load control (n = 3). Representative immunoblotting of urinary albumin and β2M (n = 3**). (E)** Quantification of cortical KIM-1 expression, albumin and β2M urinary excretion. Glomerular function was assessed by **(F)** plasma urea (n = 4), **(G)** plasma creatinine (n = 4) and **(H)** creatinine clearance (n = 4). *versus naive group, P < 0.05; versus infected group, P < 0.05.

FTY720 treatment had no effect on the levels of parasitemia (**Figure 8B**) but it drastically reduced the frequency of CD4^+^ and CD8^+^ T cells in the blood, confirming the ability of FTY720 to sequester lymphocytes in the lymphoid organs (**Figures 8C, D**). To examine whether FTY720 influences renal dysfunction, we evaluated the levels of the renal injury markers at day 5 p.i. Infected mice showed an increase in 24-h proteinuria and urinary protein to creatinine ratio (UPCr, **Figures 9A, B**). Also, infection induced an increase in the levels of albumin in the urine as well as urinary γ-glutamyl transferase (γ-GT) activity, cortical kidney injury molecule-1 (KIM-1) and urinary β2-microglobulin (β2M), markers of renal tubular injury (**Figures 9C–E**). Glomerular dysfunction was also observed in infected mice as shown by increase in the concentration of plasma urea and creatine followed by decrease in the estimated glomerular filtration rate, measured by creatinine clearance (**Figures 9F–H**). Treatment of naive animals with FTY720 did not modify any of the renal parameters studied.

**Figure 10:**
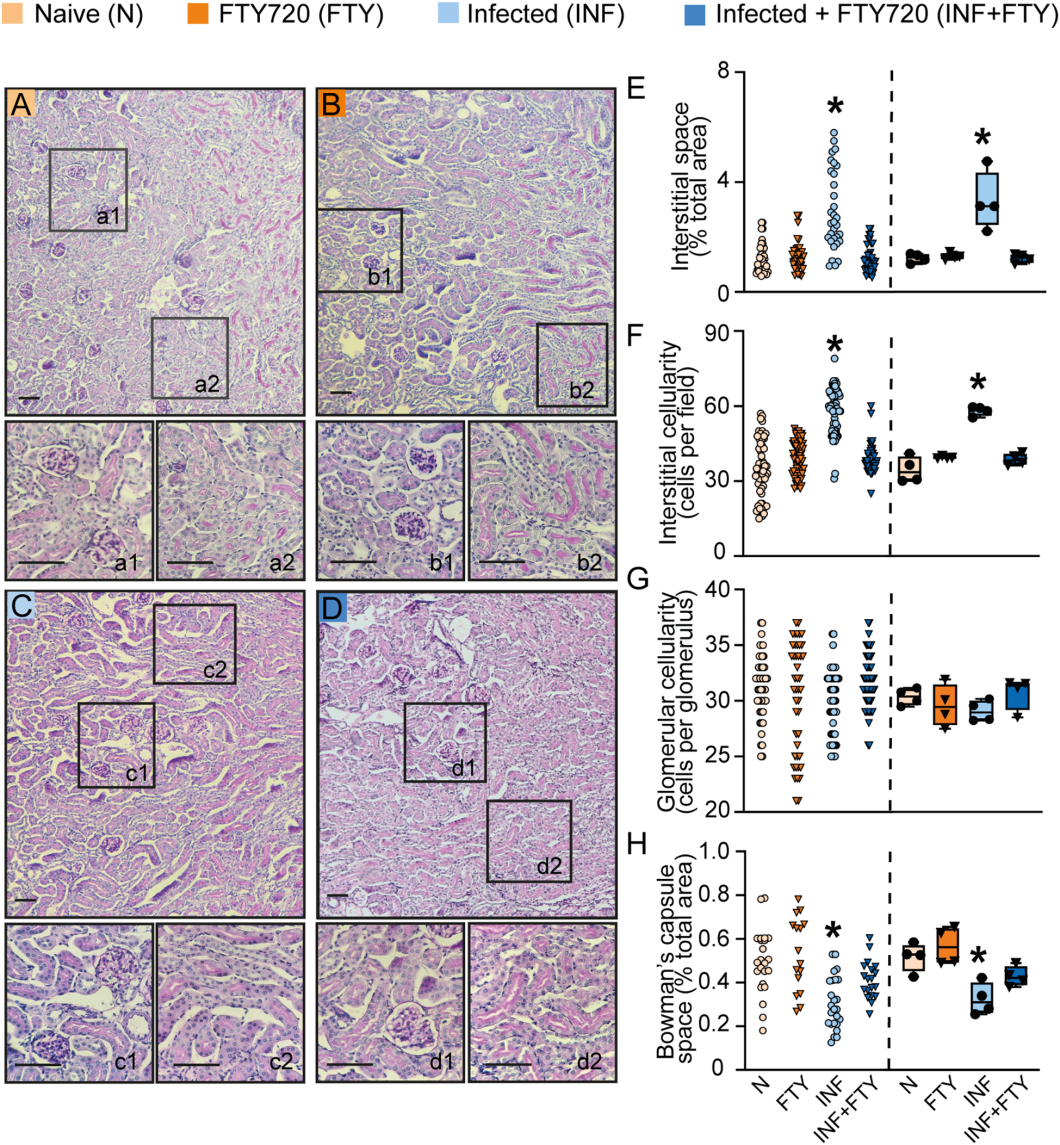
FTY720 improves tubular histomorphological parameters in PbA-infected animals. **(A–D)** Representative images of periodic acid-Schiff (PAS)-stained cortical slices of the tubular and glomerular region. Panels a1, b1, c1 and d1 display magnified views of the glomeruli, while a2, b2, c2 and d2 show magnified views of the interstitial region (scale bar, 150 μm, n = 4). **(E)** Graph of interstitial space quantification. **(F)** Graph of interstitial cellularity quantification. **(G)** Graph of glomerular cellularity quantification. **(H)** Graph of Bowman’s capsule space quantification. The dispersion plots in graphs **(E–H)** (on the left) represent the number of fields analyzed in the tubular and glomerular regions, while the box plots (on the right) show the mean values derived from the different fields for each animal (n = 4). Statistics were performed based on the average value of each animal analyzed. *versus naive group P < 0.05, versus Infected group P< 0.05.

**Figure 11:**
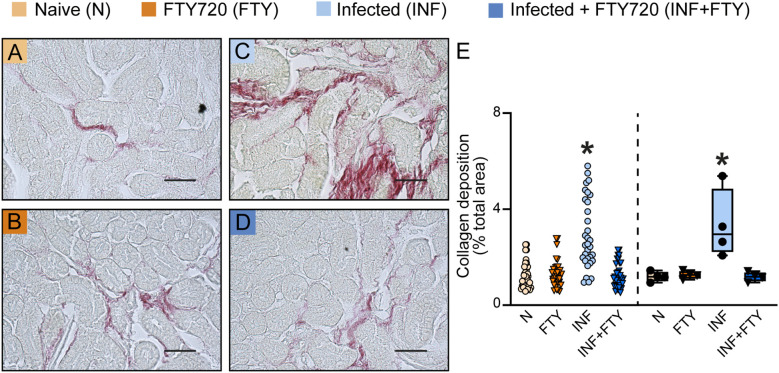
FTY720 prevents collagen deposition in PbA-infected animals. **(A–D)** Representative images of picrosirius red-stained cortical slices of the tubular and glomerular region. **(E)** Graph of collagen deposition quantification. The dispersion plot (on the left) represents the number of fields analyzed in the tubular and glomerular regions, while the box plot (on the right) shows the mean values derived from the different fields for each animal (n = 4). Statistics were performed based on the average value of each animal. *versus Naïve group P < 0.05.

To confirm the renal effects of FTY720 treatment in malaria, we evaluated several morphofunctional parameters (**Figure 10**). Infected animals treated with FTY720 exhibited a reduction in both interstitial space and cellularity (**Figures 10D, d1, E, F**) compared to infected, untreated animals (**Figures 10C, c1, E, F**). While glomerular cellularity remained unchanged (**Figures 10C, c2, D, d2, G**), the reduced Bowman’s capsule space in infected animals was restored to control levels following FTY720 treatment (**Figures 10C, c2, D, d2, H**). Additionally, we assessed collagen deposition (**Figure 11**). Infection caused significant interstitial collagen accumulation (**Figures 11C, E**), but this effect was mitigated by FTY720 treatment (**Figures 11D, E**).

Our results suggest that infection with PbA induces both glomerular and tubular damage, characteristic of MAKI. However, treatment with FTY720 specifically prevented tubular injury, suggesting a specific role for T cells in this process.

## 4 Discussion

MAKI has emerged as an important clinical complication in patients with severe malaria, and its occurrence has been consistently associated with an increased risk of mortality (Conroy et al., 2016, 2019; Oshomah-Bello et al., 2020). Using the ECM model, we demonstrated an important role of a CD8^+^ T cell-mounted response that directly induces tubular structural damage and dysfunction. Our findings provide new insights into the immunopathogenic mechanisms involved in the development of MAKI.

There is ongoing debate in the literature regarding whether the ECM model accurately represents the disease in humans. Strangward and colleagues identified common features in the pathogenesis of both diseases, including the accumulation of infected erythrocytes in the brain, occlusion of brain capillaries and the colocalization of cerebrovascular CD8^+^ T cells with infected erythrocytes, which correlates with the development of widespread vascular leakage (Strangward et al., 2017). Therefore, when used appropriately, ECM resembles human malaria and is considered an important tool to understand it. Like human disease, the ECM model develops multiple pathologies, including MAKI. The development of kidney disease in patients with malaria or in the animal model involves injury in both glomerular and tubular structures, accompanied by a strong proinflammatory response (Pulido-Méndez et al., 2006; Herbert et al., 2015; Souza et al., 2015). However, whether the genesis of glomerular or tubular injury is correlated or not is still an open matter.

The characterization of MAKI using mouse models has been reported by our group and others, and the disease varies according to both mouse and parasite strains. C57BL/6 mice infected with *Plasmodium berghei* ANKA exhibit mesangial proliferation in the glomeruli, accompanied by increased malaria pigment deposition. Moreover, in this model, reduced urinary flow is observed as well as increased levels of plasma creatinine and blood urea nitrogen associated with a significant decrease in creatinine clearance, a marker of estimated glomerular flow rate, reinforcing glomerular injury. Tubule-interstitial injury in PbA-infected mice is characterized by high levels of urinary γ-GT, β2M and cortical KIM-1 which is associated with increased collagen deposition and expansion of interstitial space. All these changes correlate with high levels of renal proinflammatory cytokines and proteinuria in infected mice (Souza et al., 2015; Silva et al., 2018; Possemiers et al., 2022).

The exact mechanism underlying the pathogenesis of MAKI remains incompletely understood, but prevailing hypotheses attempt to explain it. These include hemodynamic perturbations, immune-mediated glomerular due to the accumulation of host monocytes within glomerular capillaries, acute tubular injury and metabolic disturbances (Barsoum, 2000; Katsoulis et al., 2021). Acute tubular injury is the most common and consistent finding in the histopathology of patients with severe malaria (Barsoum, 2000; Katsoulis et al., 2021). However, as far as it is known, there are no reports in the literature regarding T cell-mediated tubulopathy.

Using adoptive transfer protocol, we accessed the effects of T cells activated during malaria infection on renal function. We demonstrated that T cells recovered from PbA-infected mice can migrate to different organs, including the kidneys, of naive acceptor mice. Our group has previously analyzed spleen-derived T cells in infected animals and observed an increase in CD4^+^ T cells with a pro-inflammatory profile, as well as CD8^+^ T cells expressing perforin, a marker of activation (Silva-Filho et al., 2013). Once in the kidneys, T cells were able to replicate the tubular injury observed in infected mice, highlighting the key role of T cells in tubular injury and MAKI. Interestingly, adoptive transfer of T cells results in immediate renal damage, observed as early as the first day post-transfer. This rapid onset suggests that the transferred T cells are already primed to induce damage. However, despite the initial impact, the injury plateaus and does not progress over the three days of analysis. We hypothesize that this plateau may be related to the absence of continuing antigen presentation in the kidney since recipient mice were not infected. Further studies are needed to test this possibility. The definitive role of CD8+ T cells in renal tubular pathology was confirmed by antibody-driven depletion experiments, which protected mice from tubular but not glomerular damage.

Clinical studies have demonstrated a correlation between HRP2, a parasite protein used to assess parasitemia, and the extent of renal injury in malaria patients. Additionally, a correlation between suPAR, a marker of the host’s inflammatory response, and the stage of renal injury has been observed, indicating that both the presence of the parasite and the host’s immune response play critical roles in the progression of renal injury (Plewes et al., 2014). These findings suggest that while the adoptively transferred T cells can initiate renal damage, the progression of this injury appears to depend on the presence of the parasite and sustained immune activation.

Although the role of CD8^+^ T cells in the genesis of MAKI is unknown, several studies have implicated them in kidney injury across different models. In cisplatin-induced kidney injury, animals lacking T cells have less renal tubular damage, and T cell replacement causes the injury to return. Moreover, cisplatin-treated CD8-deficient mice showed improved survival and renal function compared with wild-type mice (Liu et al., 2006). Linke et al., using a murine model of lupus nephritis, showed CD8^+^ T cells in close contact with proximal tubules (Linke et al., 2022). The group provided evidence of antigen cross-presentation induced by proximal tubule cells responsible for inducing and sustaining CD8^+^ T cell activation and cytotoxic function, suggesting a role in disease pathology.

During malaria infection, infected erythrocytes sequester in the endothelium or other erythrocytes, inducing endothelial activation, cytokine production and renal tissue hypoxia (Brown et al., 2020; Katsoulis et al., 2021). Sadler et al. recently demonstrated the importance of IFN-γ in infection-induced renal injury in animals infected with *Plasmodium berghei* NK65. Notably, IFN-γ knockout mice exhibited reduced glomerular and tubulointerstitial injury (Sadler et al., 2025). Accordingly, we found increasing levels of IFN-γ, IL-6, and IL-17, all known to contribute to renal disease pathology in different models (Peruchetti et al., 2020; Teixeira et al., 2020). IFN-γ has a central role in the activation of brain endothelial cells, increasing the expression of adhesion molecules such as ICAM-1 and VCAM-1 as well as leukocyte recruitment to the brain of PbA-infected mice (Swanson et al., 2016). In acute lung injury, IFN-γ contributes to activation of lung endothelial cells and cross-presentation of PbA antigens, perpetuating CD8^+^ T cell activation and lung damage (Claser et al., 2019). The mechanism of cross-presentation by renal tubular cells has been described, but additional experiments are necessary to explore this further, which is beyond the scope of the present work.

At least at the level of gene expression, we observed that *Plasmodium chabaudi* recapitulates several injury characteristics seen in PbA-induced MAKI. Both models elicit glomerular and tubular impairment, along with a pro-inflammatory response and a notable increase in the cytotoxic response (Lloyd et al., 1993; Chang and Stevenson, 2004; Silva-Filho et al., 2013; Yashima et al., 2017; Ramos et al., 2019). These findings suggest that the renal effects induced by PbA infection are not exclusive to this model and can be translated in other MAKI models. In accordance, we compared Pcc-induced MAKI with RNAseq data from different kidney injury models bearing similarities with MAKI development. Our analysis revealed a positive correlation between MAKI and the injury profiles induced by rhabdomyolysis and endotoxemia. This transcriptional overlap could suggest non-specific activation of CD8^+^ T cell responses rather than activation by parasite-specific antigens. Our results cannot rule out this hypothesis.

Rhabdomyolysis and endotoxemia are associated with increased renal hemeprotein and elevated plasma antigens, respectively, similar to malaria (Opal et al., 1999; Zorova et al., 2016; Ramos et al., 2019; Martiáñez-Vendrell et al., 2020). Plewes and colleagues demonstrated that blocking the oxidative damage caused by free heme in *Plasmodium falciparum*-infected patients reduced the kidney injury, as indicated by reduced levels of plasma creatinine (Plewes et al., 2018). Also, a correlation between suPAR (soluble urokinase-type plasminogen activator receptor), a marker of immune response, and plasma creatinine and urinary NGAL, which are markers of glomerular and tubular injury, respectively, has been demonstrated (Plewes et al., 2014). This suggests that the elevation of free heme and plasma antigens may also contribute to the development of malaria-induced kidney injury.

Conversely, no correlation was found between the IRI model and MAKI suggesting that ischemia of renal tissue induced by renal microvascular obstruction may not be as relevant for the development of MAKI. Supporting this hypothesis, recent evidence using PbNK65 knockout for skeleton binding protein-1, a protein proposed to promote the cytoadhesion of infected erythrocytes to endothelial cells, showed no improvement in renal injury compared to wild-type PbNK65-infected mice (Possemiers et al., 2022).

Our results highlight the distinct contributions of glomerular and tubular compartments to MAKI, prompting the question of why these effects differ. The current explanation relies on the specific expression of proteins in each renal compartment, such as heme oxygenase-1 (HO-1). During malaria infection, a significant increase in tubular HO-1 expression occurs, which protects tubular segments from oxidative damage induced by hemoglobin and free heme. Interestingly, this phenomenon does not occur in the glomerulus, making it a target for oxidative damage, and innate and humoral response (Ramos et al., 2019; Grunenwald et al., 2021). So, it seems that renal control of disease tolerance to malaria is compartment specific. In the present work, we demonstrated that blocking T cell migration with FTY720 particularly prevented tubular damage but had no effect in glomerular injury. Similarly, in a model of kidney disease induced by ischemia and reperfusion, it was reported that treatment with FTY720 prevented tubular injury and ameliorated the unrecoverable acute renal failure induced by ischemic injury (Oshomah-Bello et al., 2020). These results complement our observations using adoptive transfer of malaria-activated T cells, which induced tubular injury without causing glomerular alteration.

Malaria causes damage to both the glomeruli and renal tubules. However, we demonstrate that tubular injury is immunomediated by CD8+ T cells, while glomerular damage is likely driven by parasite-related mechanisms such as cytoadherence of infected erythrocytes and immune complex deposition (Katsoulis et al., 2021). This has clinical implications, as the main parameters used to assess kidney function—plasma creatinine, eGFR, and BUN—primarily reflect glomerular function, neglecting tubular injury. Moreover, current malaria treatment focuses on eliminating the parasite (WHO Guidelines for malaria, 2024) without addressing infection-induced immunopathology, potentially leaving recovered patients vulnerable to long-term renal sequelae.

In this study, we observed that mice receiving T cells from infected animals developed sustained tubular injury for three days, even in the absence of the parasite. This suggests that patients who recover from infection may continue to experience silent kidney damage. Clinical evidence supports this hypothesis: children who experience MAKI have a higher risk of post-discharge complications and mortality (Conroy et al., 2019), and elevated levels of NGAL, a marker of tubular injury, have been associated with the development of acute kidney disease after severe malaria recovery (Namazzi et al., 2022).

It is well established that AKI can progress to chronic kidney disease. In this context, proteinuria is an independent risk factor for the progression of renal disease. We observed that CD8^+^ T cells induced tubular proteinuria, which was efficiently prevented by treatment with FTY720. This observation could indicate that protein reabsorption mechanisms were preserved. In accordance with our findings, the effect of FTY720 in attenuating proteinuria and tubular interstitial injury has been observed in chronic kidney disease and AKI animal models (Ni et al., 2013; Xu et al., 2014). Since the severity of AKI correlates with the level of proteinuria (Han et al., 2014) and AKI in severe malaria is associated with substantial post-discharge morbidity and long-term risk of chronic kidney disease (Batte et al., 2021), it is plausible to postulate that ameliorating proteinuria could be a crucial strategy to prevent undesirable outcomes in patients with severe malaria.

In conclusion, our results demonstrated that CD8^+^ T cells play a key role in the development of MAKI, participating specifically in the development of tubule-interstitial injury. It is still unclear why these CD8+ T cells migrate to the kidney and how they induce tubular damage, and further studies are needed to better understand these mechanisms. However, modulating the host immune response to mitigate the detrimental role of CD8+ T cells could open new avenues for developing therapeutic strategies that target this pathogenic pathway, potentially offering a means to treat or even prevent malaria-induced acute kidney injury.

## Data Availability

The original contributions presented in the study are included in the article. Further inquiries can be directed to the corresponding author. Requests to access the datasets should be directed to AASP, acacia@biof.ufrj.br.

## Supplementary material

Supplementary Figure 1 Malaria-responsive T cells migrate to target organs of infection. Analysis of CD3+/CFSE cell migration to **(A)** lung, **(B)** spleen and **(C)** brain. (n = 4) *versus naive → naive group, P < 0.05.

Supplementary Figure 2 Malaria-responsive T cells induce an increase in renal proinflammatory cytokines. Renal levels of **(A)** IL-17, **(B)** IL-6, **(C)** IFN-γ, **(D)** TNF and **(E)** IL-4 were quantified and expressed corrected for total protein concentration. The number of animals (n) in each condition was 7 **(A-E)**. **(F)** The percentage increase in cytokines in relation to controls. P < 0.05 were considered significant.

Supplementary Figure 3 Adoptive transfer does not change T cell frequency in peripheral blood. **(A)** Representative histogram of CD4^+^ and CD8^+^ T cell frequency in the peripheral blood of N→N (T cells from naïve donors into naïve recipients) and I→N (T cells from infected donors into naïve recipients) groups. **(B, C)** Quantification of CD4^+^ and CD8^+^ T cells in peripheral blood (n = 3). **(D, E)** Kidney-to-blood ratio of CD4^+^ and CD8^+^ T cell frequency (n = 3). *versus naive → naive group, P < 0.05.

Supplementary Figure 4 Gating strategy used for flow cytometry analyses. **(A)** Lymphocyte population was initially gated based on SSC and FSC parameters. **(B)** CD45^+^ cells were gated and used for: **(C)** analysis of CD4^+^ and CD8^+^ T cell frequency in the kidney and spleen of CD8^+^ T cell-depleted animals; and **(F)** analysis of CD4^+^ and CD8^+^ T cells in the peripheral blood of FTY720-treated animals. **(D)** CD3^+^ cells were gated and used for: **(E)** analysis of CD4^+^ and CD8^+^ T cell frequency in the kidneys of mice that received adoptive T cell transfer; **(G–J)** analysis of the migration of CFSE-labeled adoptively transferred T cells. Representative histograms of T cell migration to the **(G)** lung, **(H)** spleen, **(I)** brain, and **(J)** kidney.
