# Supplementary figures and images for: CD8+ T cells promote tubule-interstitial damage in malaria-induced acute kidney injury
Source: Front Cell Infect Microbiol. 2025 Jun 30;15:1561806. doi: 10.3389/fcimb.2025.1561806 (PMC12257197; doi:10.3389/fcimb.2025.1561806)

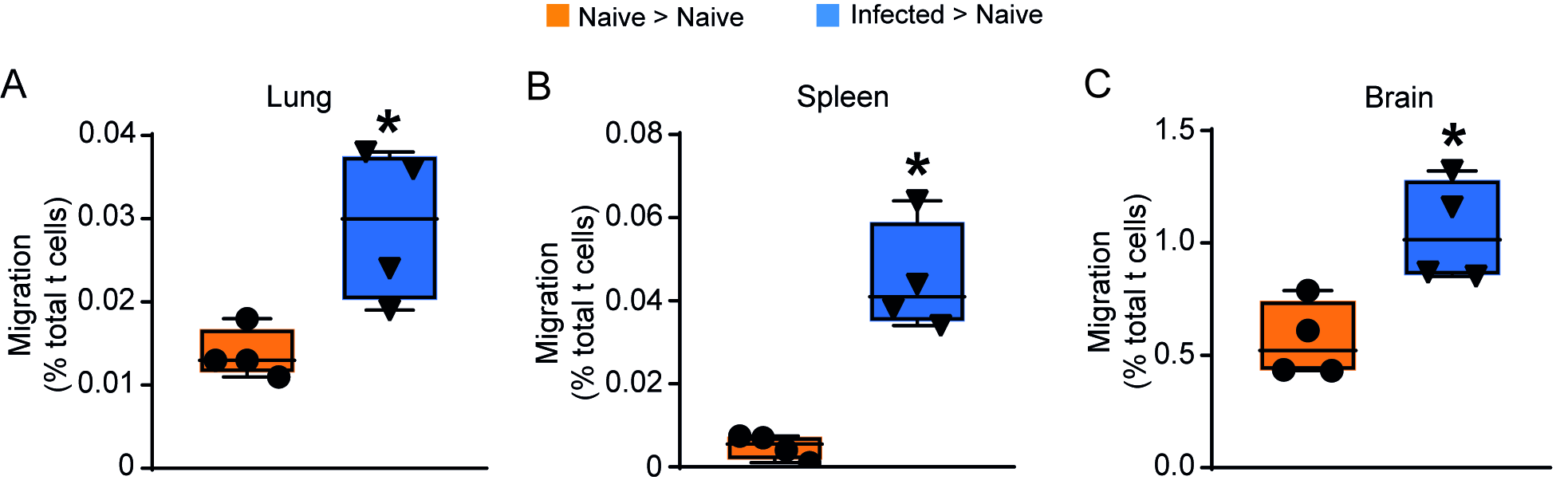

Supplement: Supplementary Figure 1 — Malaria-responsive T cells migrate to target organs of infection. Analysis of CD3+/CFSE cell migration to (A) lung, (B) spleen and (C) brain. (n = 4) *versus naive → naive group, P < 0.05. [file Image1.tif]

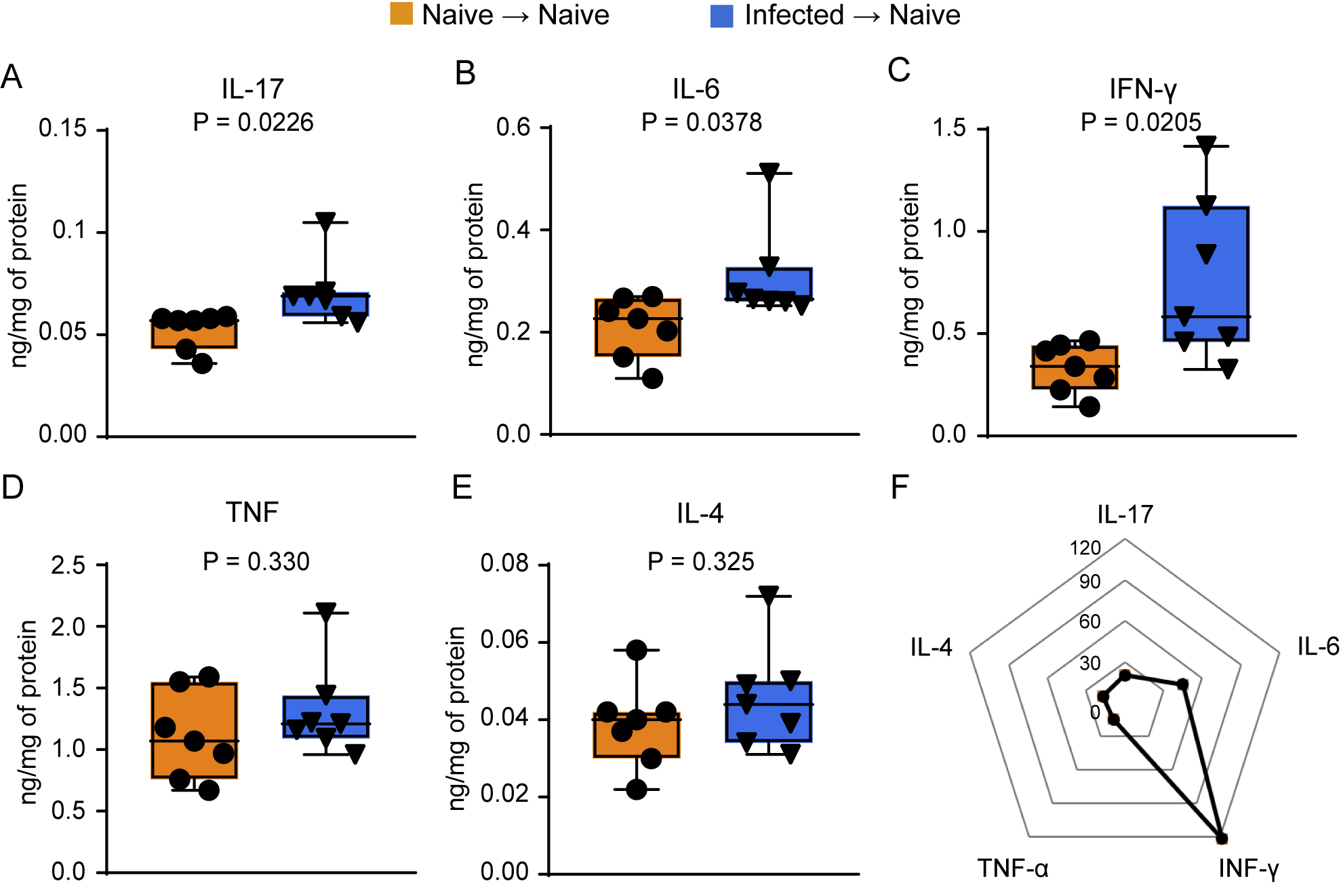

Supplement: Supplementary Figure 2 — Malaria-responsive T cells induce an increase in renal proinflammatory cytokines. Renal levels of (A) IL-17, (B) IL-6, (C) IFN-γ, (D) TNF and (E) IL-4 were quantified and expressed corrected for total protein concentration. The number of animals (n) in each condition was 7 (A-E). (F) The percentage increase in cytokines in relation to controls. P < 0.05 were considered significant. [file Image2.tif]

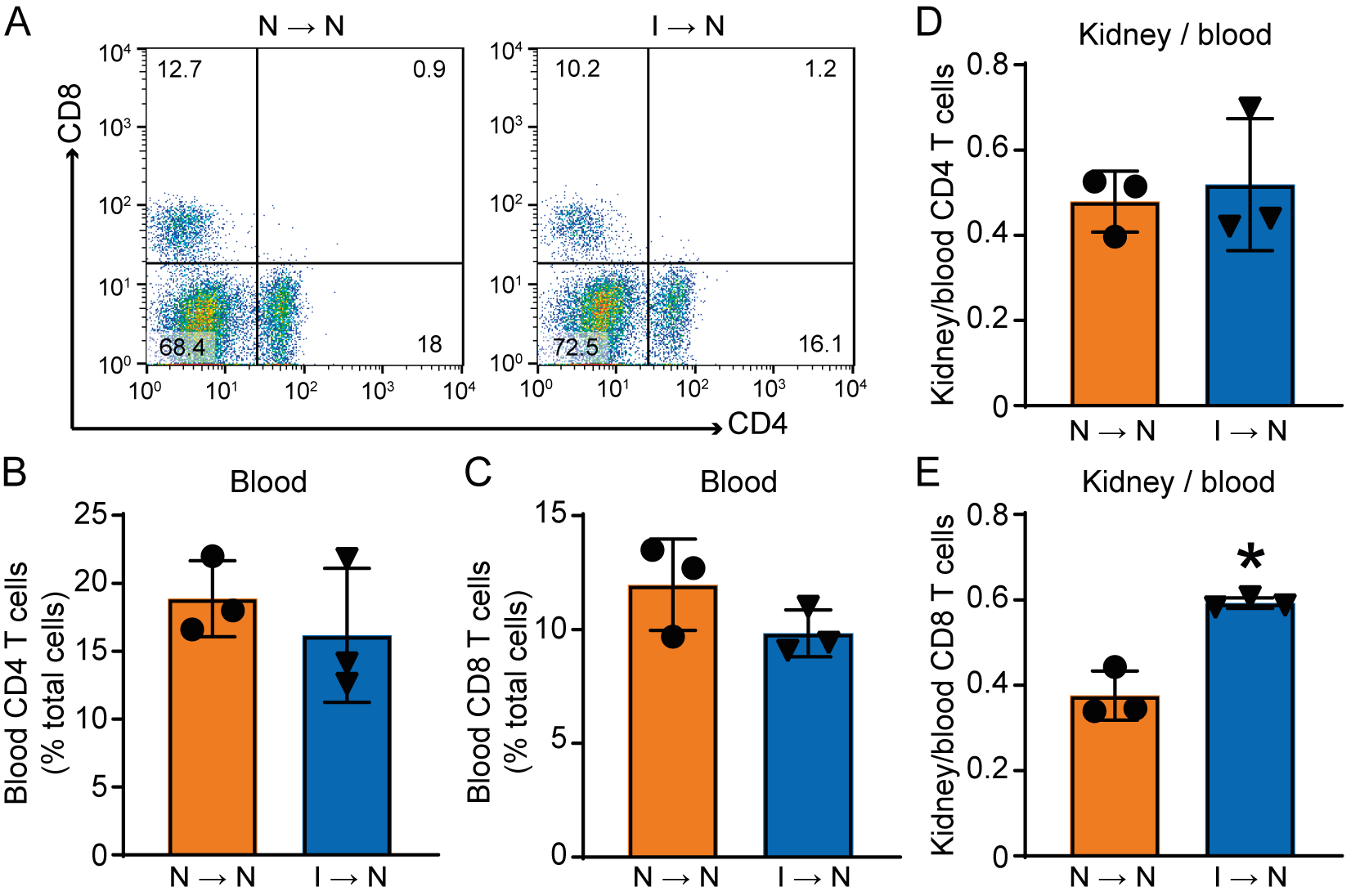

Supplement: Supplementary Figure 3 — Adoptive transfer does not change T cell frequency in peripheral blood. (A) Representative histogram of CD4+ and CD8+ T cell frequency in the peripheral blood of N→N (T cells from naïve donors into naïve recipients) and I→N (T cells from infected donors into naïve recipients) groups. (B, C) Quantification of CD4+ and CD8+ T cells in peripheral blood (n = 3). (D, E) Kidney-to-blood ratio of CD4+ and CD8+ T cell frequency (n = 3). *versus naive → naive group, P < 0.05. [file Image3.tif]

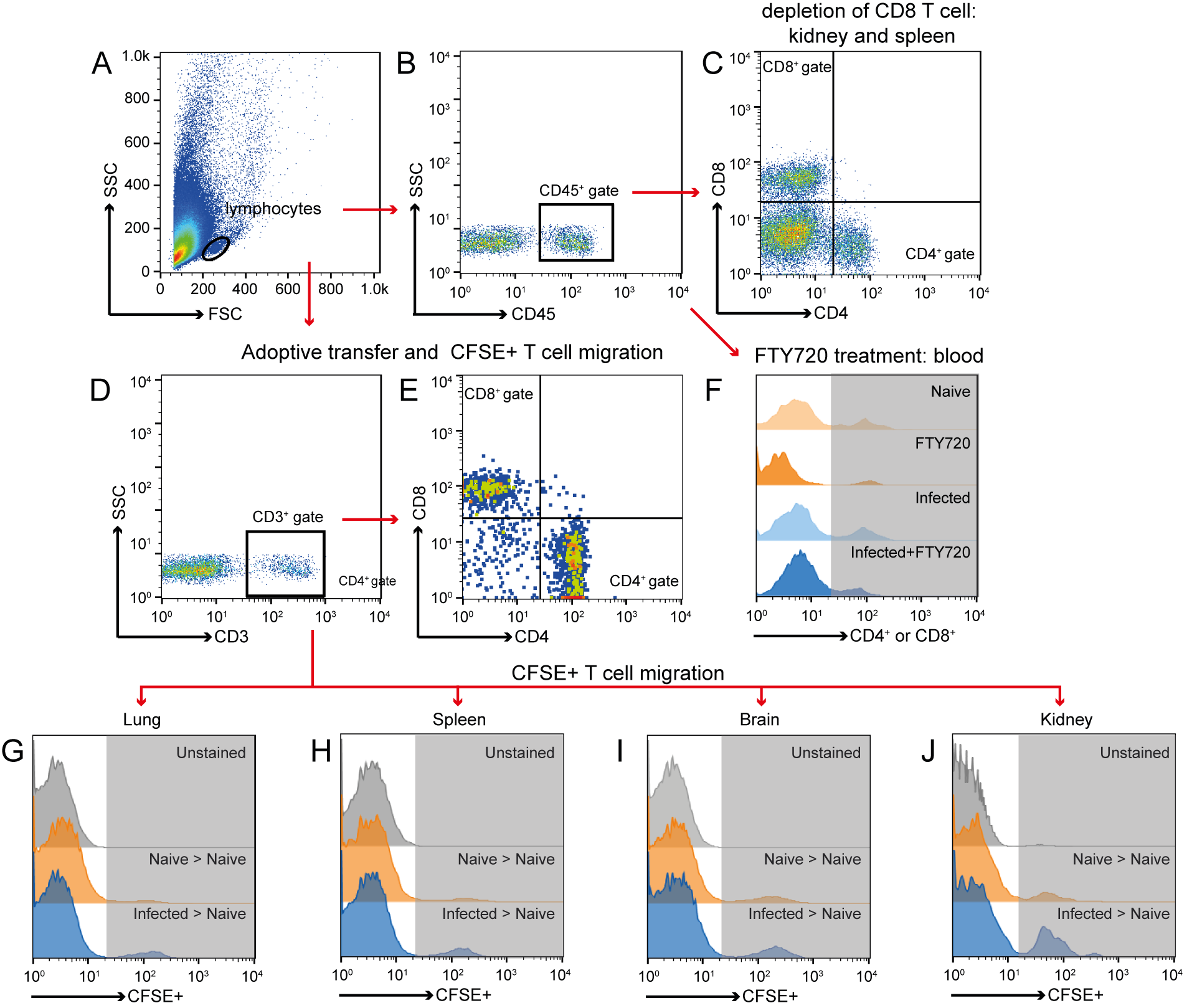

Supplement: Supplementary Figure 4 — Gating strategy used for flow cytometry analyses. (A) Lymphocyte population was initially gated based on SSC and FSC parameters. (B) CD45+ cells were gated and used for: (C) analysis of CD4+ and CD8+ T cell frequency in the kidney and spleen of CD8+ T cell-depleted animals; and (F) analysis of CD4+ and CD8+ T cells in the peripheral blood of FTY720-treated animals. (D) CD3+ cells were gated and used for: (E) analysis of CD4+ and CD8+ T cell frequency in the kidneys of mice that received adoptive T cell transfer; (G–J) analysis of the migration of CFSE-labeled adoptively transferred T cells. Representative histograms of T cell migration to the (G) lung, (H) spleen, (I) brain, and (J) kidney. [file Image4.tif]
